# Histological Sampling of Endometrial Tissue: Comparison between the MedGyn® Endosampler and Formal Fractional Curettage in Patients with Abnormal Uterine Bleeding

**DOI:** 10.31557/APJCP.2019.20.11.3527

**Published:** 2019

**Authors:** Sirichoke Tumrongkunagon, Wineeya Suknikhom

**Affiliations:** *Department of Obstetrics and Gynecology, Phrapokklao Hospital, Thailand. *

**Keywords:** MedGyn® endosampler, fractional curettage, endometrial tissue quality, uterine bleeding

## Abstract

**Background:**

Several methods have been used for evaluation of the endometrial pathology in patients with abnormal uterine bleeding. Endometrial biopsy is one of the primary methods used for diagnostic evaluation, which is a minimally invasive approach. The aim of this study was to compare the adequacy yield of samples obtained by MedGyn® Endosampler with Formal Fractional Curettage.

**Objective:**

This study was designed to compare the endometrial tissue quality and diagnostic accuracy between MedGyn® Endosampler and Formal Fractional Curettage in patients with abnormal uterine bleeding.

**Methods:**

A total of 85 endometrial tissue samples were tested by the MedGyn® Endosampler, followed by Formal Fractional Curettage, from patients at Phrapokklao Hospital who were eligible for the study and met the inclusion criteria for uterine curettage. Samples were collected between August, 2018 and April, 2019. Both operations were performed by the same investigator. The samples were submitted separately and sent to the same pathologist.

**Results:**

The mean age of the patients was 46.92 ± 6.94 years. 91.76% (78/85) of the samples obtained by Formal Fractional Curettage and 89.41% (76/85) of the samples obtained by MedGyn® Endosampler device were adequate for histopathological examination. The difference was not statistically significant (p = 0.317). The pathological results of endometrial tissue from both techniques were the same in 67 patients (78.82%) and different in 14 patients (16.47%). MedGyn® Endosampler was six times more cost effective when compared to Formal Fractional Curettage.

**Conclusions:**

Endometrial sampling using MedGyn® Endosampler is a safe, adequate, accurate, cost effective outpatient procedure which precludes general anesthesia. Therefore, it could be an alternative method for endometrial sampling.

## Introduction

Abnormal uterine bleeding is one of the most common gynecological problems, which is defined as any type of bleeding for individual patient (Sun et al., 2018). The majority present with heavy menstrual bleeding, intermenstrual bleeding, or postmenopausal bleeding that ultimately results in diagnosis. About 9-14 percent of patients with abnormal uterine bleeding causes significant morbidity, distress, work absence and restrictions on activities of daily living (Cooper et al., 2014; Narice et al., 2018). The evaluation of abnormal uterine bleeding in patients over 35 years of age or menopause is critical importance to confirm the benign nature of the problem as well as to exclude intrauterine pathology, especially endometrial carcinoma (Dimitriu et al., 2018).

Statistics in 2012 from the World Health Organization (WHO) reported that the incidence of endometrial carcinoma was 8.2 per hundred thousand people, which was the fifth highest form of cancer found in women after breast cancer, colon cancer, cervical cancer, and lung cancer. Further, the mortality rate was 1.8 per hundred thousand people.

From the same report, it was found that Thailand had incidence of endometrial carcinoma accounting for 3.9 per hundred thousand people, which was the ninth highest level in Thai women and the third highest level in the cancer of genital organs. The mortality rate in Thailand was 1.1 per hundred thousand people (Khazaei et al., 2018), which was close to the world population.

There are various methods for endometrial assessment. Formal Fractional Curettage (F/C) has been widely considered to be the gold standard for obtaining endometrial samples in microscopic evaluation. On the other hand, the need for admission, general anesthesia and the risks of perforation and haemorrhage, including the cost effectiveness, has made this option less favorable (Narice et al., 2018). Nowadays, several new and simple methods have been designed for endometrial sampling. Numerous devices for outpatients are available at present and comprise an effective and acceptable method to obtaining endometrial samples for histopathological assessment (Rodriguez et al., 1993; Inal et al., 2017), such as Pipelle, Z-sampler, Vabra aspirator (Narice et al., 2018). Moreover, approximately 10% may yield insufficient tissue for definite pathological diagnosis due to limitation of the instrument. (Aue-aungkul et al., 2018).

**Figure 1:**
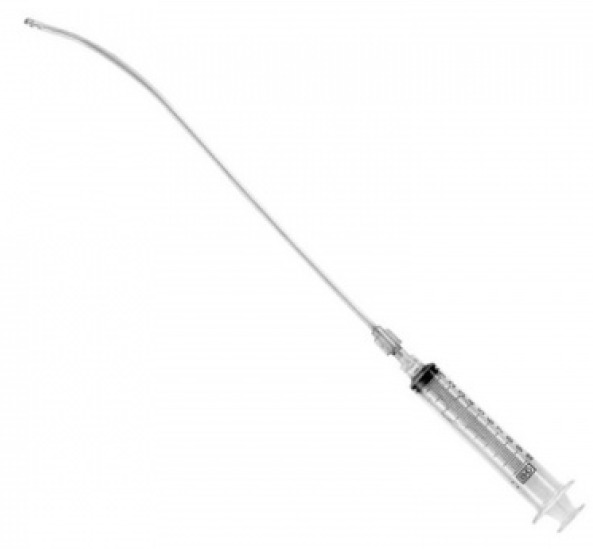
MedGyn® Endosampler

In our hospital, the only available sampling method performed at the outpatient department is the MedGyn® Endosampler (Figure 1). This device uses low-pressure suction with a semi-rigid 3 mm curette and a single sharp slot on the end (Zutshi et al., 2018) to aspirate the endometrium by utilizing a syringe technique for endometrial collection. Additionally, the device is sterile and disposable. The advantage of using an endosampler over a formal curettage includes the convenience for patients and physicians, lower cost, and less complications. Thus, study was conducted to compare the effectiveness of the MedGyn® Endosampler with the Formal Fractional Curettage technique to obtain adequate endometrial samples that provide specific and informative details for histopathologic diagnosis.

## Materials and Methods

This prospective study was conducted at the Department of Obstetrics and Gynecology, Phrapokklao Hospital (PPK Hospital) between August, 2018 and April, 2019. The study protocol and consent form were reviewed and approved by the Ethics Committee for Research on Humans in Chanthaburi Province, document ID number CTIREC-024. Women older than 35 years who presented with abnormal uterine bleeding and visited the gynecological outpatient department were included in this study. Conversely, patients who experienced vaginal bleeding related to pregnancy, coagulopathy, pelvic inflammation or infection, had cervical stenosis, lacked understanding of Thai, had a history of allergy to pethidine or diazepam, or refused to participate were excluded. 

 Clinical evaluation was done by a detailed history and clinical examination. Baseline investigations and a pelvic ultrasound were performed and informed written consent was gained prior to participation. 

$n={\left[\frac{\mathrm{za}\sqrt[{2}]{p_{0}q_{0}}+\mathrm{z\beta}\sqrt[{2}]{p_{1}q_{1}}}{p_{0}-p_{1}}\right]}^{2}$

The sample size was calculated by the test of significance of one proportion formula (Fleiss et al., 2013). According to previous studies of Khalid and Burke, (2014) and Abdelazim et al., (2013) the value of P0 = 0.96 and P1 = 0.89 were used and type I error as 5% (Zα = 1.96) and type II error as 20% (Zβ = 0.84) were determined. (q0 = 1- P0, q0 = 0.04)(q1 = 1- P1, q1 = 0.11)

$n={\left[\frac{1.96\sqrt[{2}]{0.69\times 0.04}+0.84\sqrt[{2}]{0.89\times 0.11}}{0.96-0.89}\right]}^{2}$

n=83.6

**Figure 2:**
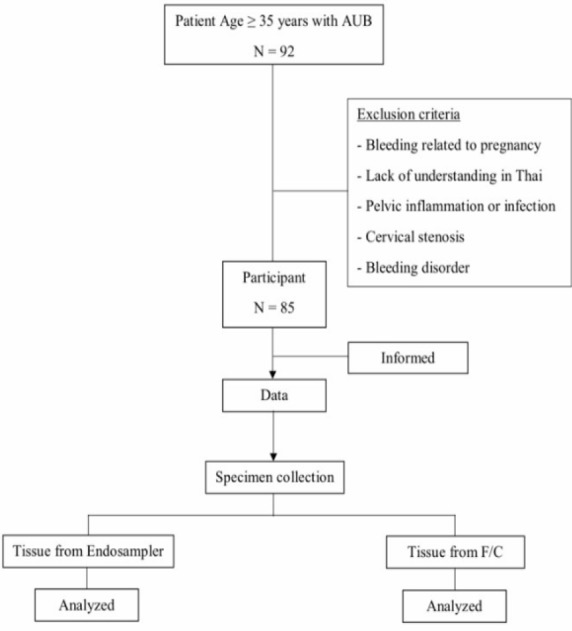
Study Flow

A total of 85 patients fulfilling the inclusion criteria were enrolled (Figure 2).

Pethidine 50 mg and diazepam 10 mg were intravenously administered 15 minutes before the procedure was started. Endometrial sampling was performed by the disposable endometrial aspiration (MedGyn® Endosampler, MedGyn, IL, USA) device in a minor operation room by obstetrics and gynecology residents. The device was introduced through the cervical canal into the uterine cavity and withdrawn outside with rotatory movement to get the sample. If the sample was insufficient, the process was repeated once or twice. The samples were collected in a container labeled as sample A. This was followed by Formal Fractional Curettage. The obtained samples from endometrial curettage were collected in a container labeled as sample B. Both samples were sent to the same pathologist. All subsequent reports contained a comment on the adequacy of the specimen, which was defined as the presence of intact endometrial glands and stroma as observed by microscopic examination, and then a diagnosis was conducted.

This study was registered at Clinicaltrials.in.th under number TCTR20191007002.

*Statistical analysis*

All data analysis was performed by Stata software and described using mean, median, and percentage. Analysis was carried out with McNemar Chi-square for comparison of the rate of adequacy. A P-value of <0.05 was considered statistically significant.

## Results

The analysis of data was performed on 85 cases whose presenting symptoms included hypermenorrhea (n=16), intermenstrual bleeding (n=50), and postmenopausal bleeding (n=19). The mean age of the study group was 46.92 ± 6.94 years in a range from 35 to 76 years. The mean parity was 1.73 ± 0.99. The mean depth of endometrium was 8.85 ± 1.82 cm and mean endometrial thickness was 0.92 ± 0.63 cm (Table1). Of the 85 endometrial samples, 91.76% (78/85) of the samples obtained by Formal Fractional curettage and 89.41% (76/85) of the samples obtained by disposable endometrial aspiration device were adequate for histopathological examination. The difference was not statistically significant (P-value >0.05) (Table 2)

The histopathological examination results for 85 samples obtained by MedGyn® Endosampler and Formal Fractional Curettage are revealed in Table 3. Finally, the cost of two method devices was compared. And it was six times more cost effective when compared to Formal Fractional Curettage.

**Table 1:** Baseline Characteristicsa

	Mean±SD	Median (range)
Age (years)	46.92±6.94	47(35-76)
Age of menarche (years)	14.07±2.00	14(11-18)
Parity	1.73±0.99	2(0-5)
Uterine depth (cm)	8.85±1.82	8(5-15)
Endometrial thickness (cm)	0.92±0.63	0.71(0.2-3.73)

**Table 2:** Tissue Adequacy Reports

Fractional Curettage	Endosampler readings (n)		Total
readings (n)	Poor quality	Good quality	
Poor quality	6	1	7
Good quality	3	75	78
Total	9	76	85

*(p=0.317) McNemar Chi-Square test

**Table 3:** Histopathological Results

Histopathology	Endosampler		Fractional curettage	
	(n)	(%)	(n)	(%)
Proliferative endometrium	41	48.2	34	40
Secretory endometrium	14	16.4	14	16.5
Hormonal effect	6	7.1	6	7.1
Endometrial polyp	6	7.1	13	15.3
Inactive endometrium	3	3.5	3	3.5
Atrophic endometrium	1	1.2	1	1.2
Endometritis	0	0.0	0	0.0
Submucous leiomyoma	0	0.0	1	1.2
Simple hyperplasia	1	1.2	2	2.3
Complex hyperplasia	0	0.0	0	0.0
Atypical hyperplasia	1	1.2	1	1.2
Adenocarcinoma	3	3.5	3	3.5
Inadequate tissue	9	10.6	7	8.2
Total	85	100	85	100

**Table 4:** Histopathological Comparison (n=85)

EM biopsy Cutettage	Normal endometrium	Polyp	Submucous leiomyoma	Endometrial hyperplasia	Adenocarcinoma	Inadequate	Total
Normal	53	3	-	-	-	1	57
endometrium							
Polyp	10	3	-	-	-	-	13
Submucous	1	-	-	-	-	-	1
leiomyoma							
Endometrial	-	-	-	2	-	1	3
hyperplasia							
Adenocarcinoma	-	-	-	-	3	-	3
Inadequate	2	-	-	-	-	6	8
Total	66	6	0	2	3	8	85

## Discussion

The main reason for performing endometrial biopsy in women with abnormal uterine bleeding was to confirm the benign nature by ruling out endometrial pathology, especially endometrial carcinoma and other indications, which remain some of the most commonly performed gynecological procedures (Rodriguez et al., 1993; Nagele et al., 1996; Inal et al., 2017; Kolhe, 2018). Medical or conservative surgery should be a choice and unneeded radical surgery should be able to be avoided. Endometrial sampling is a minimally invasive alternative method to collect endometrial tissue instead of curettage. Though various sampling methods exist, the Pipelle device is ideal for obtaining endometrial sample in outpatient department (Ilavarasi et al., 2019). Many studies have reported that the Pipelle has a diagnostic accuracy comparable with the standard method (Fractional curette) (Abdelazim et al.,2013; Sanam and Majid, 2015; Liu et al., 2015). In our institution, MedGyn® Endosampler is used for sampling endometrial tissue. Thus, this study was conducted to evaluate the efficacy of this device as a tool for endometrial biopsy. The present study demonstrated the rate of adequacy of endometrial specimen in the MedGyn® Endosampler was 89.41%, which is close to 91.76% for the Formal Fractional curettage, after analyzed there is no statistically significant difference (McNemar Chi-Square test p = 0.317).

Considering the endometrial histopathological reports from both techniques, it was found that there were similarities and differences in the results; 79% (67/85) and 21% (18/85), respectively. The same reports consisted of normal endometrium (proliferative endometrium, secretory endometrium, inactive endometrium, hormonal effect, atrophic endometrium), polyp, submucous leiomyoma, endometrial hyperplasia, adenocarcinoma. On the contrary, the different reports of histopathologic data show that the tissue of 10 patients obtained by Fractional Curettage report as polyp, while 1 patient reporting as submucous leiomyoma. The tissue obtained by endosampler reported as normal endometrium. This finding is compatible with the study of Forthergill et al., (1992), who found that the curettage was more accurate for detecting endometrial polyp than the endometrial sampling due to the limitation of the instrument, which means less ability to collect the polyp or solid mass. In the current study, there was one pathological report from curettage showing endometrial hyperplasia, while the endosampler could not obtain the tissue. This may be due to the pathology being only a focal lesion that prevented the device from detecting any abnormality.

In the same group of patients, one sample obtained by the endosampler could not be interpreted, but could be reported as normal endometrium when collected by curettage (Table 4). This may be due to the endometrial thickness being too thin for collection by the sampling, but enough for collection by curettage. Moreover, it may be placed into an inappropriate position. For the two samples obtained by endosampler, they reported as having normal endometrium, whereas could not report when obtained by curettage due to inadequate tissue (Table 4). This may result from all tissue being collected by endosampler. 

In order to minimize the variation among participants, a self-controlled study was chosen as the study design to compare between the two methods. Moreover, a well-organized process of tissue collection and identification was utilized. As for pathological assessment, all tissue was sent to the same pathologist to decrease interobserver variability. Therefore, the study results could more accurate and reliable.

From this study, MedGyn® Endosampler has proven to be an effective method for endometrial tissue collection with results similar to Formal Fractional Curettage. As for the pathological aspect, it also demonstrates the same results and could detect important lesions in endometrial pathology, including premalignant and malignant lesions. However, there were some limitations of the instrument when used with patients whose symptoms were caused by endometrial polyp or submucous leiomyoma. Nowadays, there are minimally invasive alternative methods used to detect endometrial polyp or submucous leiomyoma, such as three-dimensional saline infusion sonography (3D SIS) (Nieuwenhuis et al., 2017) and office hysteroscopy.

In conclusion, uterine curettage is a gold standard procedure for obtaining endometrial samples used in pathological diagnosis in the case of abnormal uterine bleeding. MedGyn® Endosampler is an outpatient procedure that precludes anesthesia along with its associated complications. Further, it is more cost effective and requires no hospitalization. Moreover, the effectiveness of tissue obtained for diagnosis showed reliable histopathology results when compared with Formal Fractional Curettage. Conclude that, this device is a good alternative method for endometrial tissue sampling.
